# Attracted to power: challenge/threat and promotion/prevention focus differentially predict the attractiveness of group power

**DOI:** 10.3389/fpsyg.2015.00397

**Published:** 2015-04-07

**Authors:** Annika Scholl, Claudia Sassenrath, Kai Sassenberg

**Affiliations:** ^1^Department of Social Psychology, Institute of Psychology and Education, University of Ulm, Ulm, Germany; ^2^School of Science, University of Tübingen, Tübingen, Germany

**Keywords:** regulatory focus, threat, challenge, groups, social power

## Abstract

Depending on their motivation, individuals prefer different group contexts for social interactions. The present research sought to provide more insight into this relationship. More specifically, we tested how challenge/threat and a promotion/prevention focus predict attraction to groups with high- or low-power. As such, we examined differential outcomes of threat and prevention focus as well as challenge and promotion focus that have often been regarded as closely related. According to regulatory focus, individuals should prefer groups that they expect to “feel right” for them to join: Low-power groups should be more attractive in a prevention (than a promotion) focus, as these groups suggest security-oriented strategies, which fit a prevention focus. High-power groups should be more attractive in a promotion (rather than a prevention) focus, as these groups are associated with promotion strategies fitting a promotion focus (Sassenberg et al., 2007). In contrast, under threat (vs. challenge), groups that allow individuals to restore their (perceived) lack of control should be preferred: Low-power groups should be less attractive under threat (than challenge) because they provide low resources which threatened individuals already perceive as insufficient and high-power groups might be more attractive under threat (than under challenge), because their high resources allow individuals to restore control. Two experiments (*N* = 140) supported these predictions. The attractiveness of a group often depends on the motivation to engage in what *fits* (i.e., prefer a group that feels right in the light of one’s regulatory focus). However, under threat the striving to *restore control* (i.e., prefer a group allowing them to change the status quo under threat vs. challenge) overrides the fit effect, which may in turn guide individuals’ behavior in social interactions.

## Introduction

Imagine you have the possibility to join a new team. You could become a member of a high-power group (e.g., a team of sports referees or a group of supervisors) that is relatively independent and has access to many resources. You could also join a low-power group (e.g., a group of sports players who depend on the referees’ decisions, or a team of subordinates continuously evaluated by their supervisors) that is more constrained and depends on others when doing their work. The group’s social power has clearly an impact on social interactions within and beyond that group. Under which conditions would these groups appear attractive for you to join?

Low-power is often less attractive than high-power (e.g., Sachdev and Bourhis, 1985, 1991; Bourhis, 1994; but see Schmid Mast and Hall, 2003), because it implies lower resource control (Keltner et al., 2003). On the one hand, this lack of control will seem especially inattractive when the demands of a situation seem excessive, such as under threat. However, on the other hand, being in a position of low control allows individuals to keep a “low profile” and to simply follow others’ lead. This might appear relatively more attractive under different conditions. Thus, we argue that the degree to which a person is attracted to a low- and a high-power group, respectively, crucially depends on that person’s *motivation*.

In this sense, we investigate the impact of two sets of distinct motivational states on the attraction to group power: a prevention vs. a promotion focus (Higgins, 1997, 1998), and a threat vs. challenge state (Blascovich and Tomaka, 1996). Previous research treated these motivational states as being closely related to each other or even overlapping, due to their focus on potential losses (in case of both a prevention focus and threat) or gains (for both a promotion focus and challenge). Indeed, research shows that a prevention focus facilitates threat reactions and that a promotion focus elicits challenge responses (e.g., Keller, 2007, 2012; Keller and Bless, 2008; Seery et al., 2009; Sassenrath et al., unpublished). Inversely, threat elicits behavior in line with a prevention focus (i.e., avoiding errors), whereas challenge evokes behavior in line with a promotion focus (i.e., using opportunities for gains; Seibt and Förster, 2004; Derks et al., 2006; Oyserman et al., 2007).

Despite these commonalities, the present research seeks to provide evidence that prevention and threat, as well as promotion and challenge, do *not* lead to the same but different evaluations of self-relevant social targets. We provide evidence that prevention-focused individuals evaluate low-power groups as being relatively more, and high-power groups as being less, attractive than promotion-focused individuals. In contrast, individuals under threat evaluate low-power groups as being even less and high-power groups as being even more attractive than when they are challenged. The current research has two goals. First, it seeks to contribute to an understanding of how these two sets of motivational states—despite their commonalities—differ in terms of their outcomes in social context. Second, it aims at highlighting how threat affects the attraction to and potentially also the choice of social settings compared to other motivational states.

### Regulatory Focus and the Attraction to Group Power

Regulatory focus theory (Higgins, 1997, 1998) posits two distinct motivational systems, which can vary chronically and situationally: The *promotion focus* refers to the regulation of the needs for nurturance, gain, and achievement. This leads to applying eager, approach-related strategies, such as ensuring “hits” and approaching ideal states. In contrast, the *prevention focus* is concerned with regulating the needs for security and safety, which results in the application of cautious strategies, such as ensuring “correct rejections” and avoiding to miss one’s obligations. In a promotion focus, events are perceived as gains vs. non-gains. In a prevention focus, events are perceived in terms of non-losses vs. losses. In short, promotion-focused individuals strive toward taking advantage of opportunities, whereas prevention-focused individuals strive toward not making mistakes.

This preference for self-regulatory strategies influences the evaluation of targets (e.g., events, behaviors, or social groups). Individuals find targets that are compatible with their regulatory focus more attractive (Higgins, 2000; see for example Higgins et al., 2003). This suggests that evaluations of groups should likewise depend on how well individuals expect the group to fit their regulatory needs: Groups that individuals expect to fit their regulatory needs should “feel right” and, thus, be more attractive to join. What does this imply for the attractiveness of a high- and a low-power group?

Social power is defined as asymmetric control over one’s own and others’ outcomes (Fiske and Berdahl, 2007). This means that *high-power groups* have control over resources (e.g., information, food, money, social appreciation) which others depend on. High-power groups have a higher capacity to modify another (i.e., a low-power) group’s state than *vice versa*. This provides those high in power with relative freedom to interact with others in a way they prefer while being independent from them; in contrast, *low-power groups* are per definition relatively dependent on others (i.e., on high-power groups; Fiske and Dépret, 1996; Keltner et al., 2003; Fiske and Berdahl, 2007). A low-power group depends more strongly on others to get access to the required resources. Examples for typical high- vs. low-power groups refer to the groups of professors/students at the university, referees/players in sports, or leaders/subordinates in organizations.^1^

In this regard, a *high*-power group allows individuals to engage in behaviors that fit the regulatory needs of nurturance and striving for gain (i.e., taking advantage of opportunities), and individuals are well aware of this: Those high in power likely display promotion-oriented behaviors (Keltner et al., 2003), and individuals likewise anticipate high-power groups to provide them with the opportunity to display promotion-focused behavior (Sassenberg et al., 2007). In other words, high-power groups not only provide individuals with resource control once they have become a group member, but individuals (i.e., non-group members) also *expect* high-power groups to provide such control. In contrast, a *low*-power group has limited access to resources and faces more social constraints. As a result, those low in power act more carefully, in a more prevention-oriented way (e.g., taking care not to make a mistake or a negative impression on the high-power group; Keltner et al., 2003). Conversely, individuals also *expect* low-power groups to allow them to demonstrate such prevention-focused behavior (Sassenberg et al., 2007).

To conclude, individuals hold *expectations* with regard to which behavior will likely be demonstrated in high- and low-power groups. As a result, regulatory focus should make groups with a different amount of power more attractive: *High*-power groups are expected to provide the opportunity to apply promotion-oriented strategies which better fit a promotion (than a prevention) focus. Consequently, high-power groups should be relatively more attractive to promotion-focused (than prevention-focused) individuals. Similarly, *low*-power groups are assumed to allow for safety- and security-oriented behaviors. These behaviors do not “feel right” in a promotion focus, but better fit a prevention focus. Hence, low-power groups should be evaluated more favorably in a prevention (than in a promotion) focus.

Empirical evidence supports this idea. Indeed, Sassenberg et al. (2007, see also Sassenberg and Scholl, 2013; Sassenberg et al., 2013) demonstrated that *promotion*-focused individuals (explicitly and implicitly) evaluate high-power groups as being more attractive than prevention-focused individuals. This effect disappears if high-power is no longer associated with opportunities—in other words, if the resulting promotion-oriented behaviors in the group become less likely (i.e., rather when the responsibilities which high-power implies are salient; Sassenberg et al., 2012), providing further support that individuals prefer groups that likely fit their regulatory focus. *Prevention*-focused individuals, in contrast, indeed evaluated low-power groups more favorably than did promotion-focused individuals. In these studies, regulatory focus predicted the attraction to group power among non-group members (i.e., when individuals did not yet belong to the group; Sassenberg et al., 2007, 2013). However, this similarly seems to apply to group-members (e.g., when individuals already belong to a high-/low-power group at work; Sassenberg and Scholl, 2013). While these studies clearly supported the predictions concerning the impact of regulatory focus and group power on group attraction, high-power groups were rated as more attractive than low-power groups in all studies assessing group attraction explicitly.

In short, this research indicates that a promotion (vs. prevention) focus makes high-power groups even more attractive, whereas a prevention (vs. promotion) focus promotes the attractiveness of low-power groups—because individuals expect that belonging to the respective group allows them to *engage in* their preferential self-regulatory behavior. Do threat and challenge, which are often considered closely related to prevention and promotion focus, result in parallel effects? As we will outline in the following, threat and challenge likely produce distinct effects on the attraction to group power—in particular, because the motivation resulting from threat counteracts the fit effect.

### Threat vs. Challenge and the Attraction to Group Power

The Biopsychosocial Model of Threat and Challenge (Blascovich and Tomaka, 1996) describes how individuals respond to goal-relevant situations that require active dealing with the demands resulting from the goal at hand (so-called motivated performance situations). Examples include situations in which individuals perform a test or give a speech in front of an audience. By definition, threat and challenge as such arise when individuals perceive a discrepancy to a desired end-state—referring to the examples above, individuals may seek to perform well on the test or to impress the audience. This discrepancy and the resulting tendency to reduce it (i.e., the striving for a change of the status quo) are not part of the two regulatory foci. This difference is crucial for the following argument.

When perceiving such a discrepancy, individuals evaluate whether their personal resources (e.g., knowledge, skills) match the situational demands (e.g., the effort required for a task). Accordingly, they respond with *threat* when personal resources fall below the demands of the current situation—that is, when individuals perceive chances to be too low to overcome the discrepancy. For instance, threat occurs when individuals perceive that the demands of a math test exceed their personal skills to solve it, or perceive that their skills to deliver a speech fall below the expectations of the audience. In contrast, if they experience personal resources to match (or exceed) the situational demands, individuals respond with *challenge*: They will likely overcome the discrepancy, even though it might be effortful. In sum, challenged individuals see a high likelihood of gain in the given situation, whereas threatened individuals perceive a high likelihood of loss (cf. Tomaka et al., 1993).

Threat and challenge guide subsequent responses: under challenge, resources seem to suffice and individuals feel ready to master the demands at hand. As an outcome, they show more efficient physiological reactions and higher task performance (than threatened individuals; e.g., Tomaka et al., 1993; Seery et al., 2010). In contrast, under threat individuals feel they cannot cope with the demands at hand and thus experience a lack of control. Consequently, they seek for means to *restore* their control (cf. Staw et al., 1981; Kamphuis et al., 2011; see also Sherman and Cohen, 2006; Sherman and Hartson, 2011 for a similar argument). These means of control restoration can directly address the source of threat (e.g., produce defensive responses toward the “threatening” test). Importantly, however, when such direct means are not available, individuals apply indirect or palliative control restoration (see Jonas et al., 2014). That is, when being threatened in one context (e.g., one’s anticipated low performance on an upcoming test), individuals seek to restore control preferably in the context in which the threat comes up. If, however, threat cannot be regulated in such a functional way, individuals switch to *another* context or domain, for example by reaffirming their values in a context or domain that is *unrelated* to the one in which the threat originally came up (e.g., one’s skills in another field or one’s relationships with friends and family). Reminding oneself of such competencies and values that do not directly address the source of the threat at hand can (at least for some time) reduce the perceived threat (even though it is not functional in the sense that it removes the source of threat; for an overview see Sherman and Hartson, 2011). Taken together, in case of those palliative responses, threat in one context affects evaluations in another context.

We propose that this palliative regulation of threat also applies to the evaluation of high- and low-power groups: under threat, individuals perceive a lack of resources to master the current discrepancy. This elicits efforts to restore control. High-power groups, by definition, provide relative resource control, whereas low-power groups lack such control and depend on others (Fiske and Berdahl, 2007); individuals are well aware of this (Sassenberg et al., 2007). We thus expected that threat (vs. challenge) in one context would affect as how attractive individuals evaluate a high- and a low-power group. A *high*-power group should be even more attractive under threat (than challenge), because such a group allows individuals to restore control (in another context) and thereby reduce the threat; this is less important under challenge when one already experiences sufficient resources. Conversely, *low*-power groups offer low resources (and even more demands). This is particularly unattractive under threat (when resources already seem low), but less unattractive under challenge (when resources seem sufficient and control restoration is less important). In sum, we expect that high-power groups might be even more attractive to threatened (than challenged) individuals, whereas low-power groups should be even less attractive for threatened (than challenged) individuals.

Importantly, our predictions imply differences in attraction to group power between *prevention focus and threat* as well as between *promotion focus and challenge*—despite the previously outlined similarities of these states. Similarly to prevention, threat addresses potential losses and security needs. Similarly to promotion, challenge is concerned with gains and nurturance needs. However, threat/prevention as well as challenge/promotion also differ in several significant aspects. A prevention or promotion focus does not rely on the perception of a discrepancy (e.g., Crowe and Higgins, 1997; Idson et al., 2000; Grant and Higgins, 2003), but they only imply a heightened preparedness for gain- or loss-signals, respectively. Individuals are attracted to opportunities to act in line with this preparedness (according to the regulatory fit hypothesis; Higgins, 2000) because they seek to *sustain their current state*—in other words, engage in what “feels right.” However, threat and challenge by definition imply the experience of a discrepancy and, thus the striving to *change* the status quo toward the desired end-state (e.g., performing well on the upcoming test). Taken together, individuals in a prevention/promotion focus are attracted to situations sustaining their state, whereas threatened/challenged individuals strive for a change of the status quo. In particular, the striving to regain control under threat should counteract the selection of fitting group that we expect to result from regulatory focus. Different from a prevention focus, threat (compared to challenge) should lead to a preference for high-power groups.

In line with the idea that the effects of prevention and threat as well as of promotion and challenge differ, Sassenberg et al. (2015) demonstrated such differences on the *attentional level*, more specifically, the processing of negative stimuli. Both prevention-focused and challenged individuals direct their attention more to negative (than neutral) stimuli, which is not the case in a promotion focus or under threat: in a prevention focus, in which individuals are more sensitive to losses, attention is directed more toward targets signaling such potential losses (i.e., here negative stimuli that fit a prevention focus, rather than a promotion focus). In contrast, attention is not directed toward negative stimuli under threat, but rather under challenge under which one perceives sufficient resources to deal with negative targets (here, negative stimuli; Sassenberg et al., 2015). While this study focused on differences between these sets of states concerning their influence on the attention to negative targets, the current research investigates differences for the *evaluation* (i.e., valence) of (social) targets, namely how attractive it seems to join a high- or a low-power group.

### The Present Research

To sum up, we predicted that regulatory focus promotes attraction to groups that provide the potential to sustain the preferred self-regulatory strategies: a high-power group is more attractive for promotion- (than prevention-) focused individuals, whereas a low-power group is less unattractive for prevention- (than promotion-) focused individuals. Threat (vs. challenge), however, promotes attraction to groups depending on the groups’ potential to restore control: a high-power group might be more attractive under threat (than challenge), whereas a low-power group should be less unattractive under challenge (than threat). Hence, under threat group preference should be determined by the extent to which a group can serve as a resource rather than by its regulatory fit to the currently preferred self-regulatory strategies.

Results supporting this prediction would extend prior research in several ways. First, the findings would contribute to an understanding of how individuals under threat regulate their state in a subsequent social context, here, by preferring a group membership that (more or less) allows for restoring control. Second, the present research extends findings on the conditions under which a low- and high-power group is (relatively) more attractive. Third and most importantly, such findings shed further light into potential differences between the two sets of motivational states—promotion/prevention focus and challenge/threat states—and show that these should be distinguished in the social context.

Two experiments tested our predictions. The procedure for measuring attraction to a low- and high-power group closely followed Sassenberg et al. (2007) and was identical for both studies. Experiment 1A focused on the impact of promotion/prevention focus in order to replicate earlier findings on attraction to a low- and a high-power group (Sassenberg et al., 2007). Implementing the identical group context and group attraction measure, Experiment 1B tested the impact of threat/challenge. Across the two studies, regulatory focus and threat/challenge were manipulated independently of the group context, in order to rule out potential demand effects.

## Experiment 1A

### Material and Methods

#### Participants and Design

Sixty undergraduates (38 female, 22 male; *M*_age_ = 22.45, SD = 2.70; range 18–31 years) participated in an experiment with a 2 (Regulatory focus: promotion vs. prevention) × 2 (Group power: high vs. low) design. Regulatory focus was manipulated between participants, whereas Group power was a repeated measures factor. Participants took part in this study on campus in exchange for some candy.

#### Procedure

Participants completed a paper–pencil questionnaire, supposedly comprising “pretest materials” for two unrelated studies. In fact, the questionnaire included (1) the regulatory focus manipulation (Friedman and Förster, 2001) and (2) the measure of attraction to a powerful and a powerless group (Sassenberg et al., 2007). To manipulate situational regulatory focus, participants lead a mouse through a maze, either toward a mouse hole to prevent it becoming an eagle’s prey (i.e., security-related strategies were activated; prevention focus condition; *N* = 30) or toward a large piece of cheese (i.e., nurturance-related strategies were activated; promotion focus condition; *N* = 30).

As “unrelated second part” of the study, participants completed questions on the “perception of groups.” Here, they were informed that the researchers were interested in their spontaneous evaluation of a group. More specifically, they read that though it can be difficult to make a general judgment of a group and its members, individuals usually have a rough idea of how others think and act. They were then instructed to think of and afterward indicate their spontaneous evaluation of a group that included “members of a large company who have high-power and average status” (high-power group) and a group that included “members of a large company who have low-power and average status” (low-power group). The order of the two groups was counterbalanced. There were no interactions with order (i.e., high-power vs. low-power group presented first; all *F*s < 1.22, *p*s > 0.275); hence, we do not further discuss this factor in the following. The exact same power manipulation has been used before (Sassenberg et al., 2007, 2012). In those articles, it produced the same results than other power manipulations and can therefore be considered as well validated.

Two items assessed attraction to each group, respectively (“If you imagine being a member of this group, how do you feel?”; 1 = *bad* to 7 = *good*; “How attractive is this group to you?”; 1 = *not at all* to 7 = *very much*; *r*(60)_high-power-group_ = 0.73, *p* < 0.001; *r*(60)_low-power-group_ = 0.77, *p* < 0.001). One item served as manipulation check for perceived power of each group (“Which of the following attributes fits to this group?” on a 8-point semantic differential from “*not at all powerful*” to “*very powerful*”). Finally, participants indicated their demographics, were thanked, debriefed, and compensated.

### Results

#### Checks

Results yielded an effect of Group power on perceived power, indicating that participants rated the low-power group as being less powerful (*M* = 2.83, SE = 0.24) than the high-power group (*M* = 7.35, SE = 0.25), *F*(1,58) = 103.95, *p* < 0.001, ${\text{η}}_{\text{p}}^{\text{2}}$ = 0.64. There was no interaction of Group power with Regulatory focus and/or Order on perceived power (all *F*s < 1, *p*s > 0.408). Hence, the manipulation of Group power was successful.

#### Attraction to Group Power

We predicted that prevention-focused participants would find the low-power group relatively more attractive than promotion-focused participants, whereas promotion-focused participants would find the high-power group even more attractive than prevention-focused participants.

A 2 (Regulatory focus: promotion vs. prevention) × 2 (Group power: low vs. high) mixed model analysis of variance with repeated measurement on the last factor yielded a main effect of Group power, *F*(1,58) = 23.81, *p* < 0.001, ${\text{η}}_{\text{p}}^{\text{2}}$ = 0.29, but not of Regulatory focus (*F* < 1). Overall, participants evaluated the high-power group as more attractive (*M* = 5.08, SE = 0.23) than the low-power-group (*M* = 3.38, SE = 0.20). This effect was qualified by the predicted Regulatory focus × Group power interaction, *F*(1,58) = 7.18, *p* = 0.010, ${\text{η}}_{\text{p}}^{\text{2}}$ = 0.11.

**FIGURE 1:**
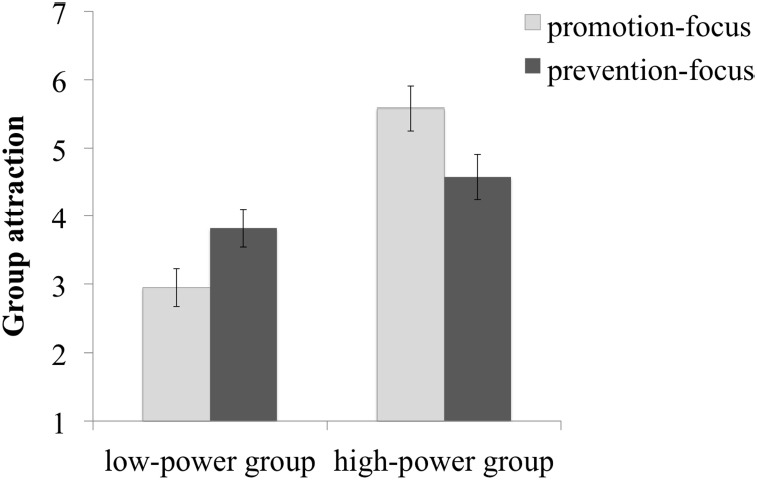
**Group attractiveness depending on group power and regulatory focus, Experiment 1A (*N* = 60)**.

The high-power group was more attractive than the low-power group in the promotion focus condition, *F*(1,58) = 28.57, *p* < 0.001, η^2^ = 0.33, but not (significantly so) in the prevention focus condition, *F*(1,58) = 2.42, *p* = 0.125, η^2^ = 0.04. More importantly, as expected, the low-power group was more attractive to prevention-focused (*M* = 3.82, SE = 0.28) than promotion-focused participants (*M* = 2.95, SE = 0.28), *F*(1,58) = 4.86, *p* = 0.031, η^2^ = 0.08, MD(mean difference) = 0.87, CI_95%_[0.08, 1.65]. In contrast, the high-power group was more attractive to promotion-focused (*M* = 5.58, SE = 0.33) than prevention-focused participants (*M* = 4.58, SE = 0.33), *F*(1,58) = 4.59, *p* = 0.036, η^2^ = 0.07, MD = 1.00, CI_95%_[0.07, 1.93], see Figure 1.

### Discussion

This experiment supported our assumptions and is in line with earlier research (Sassenberg et al., 2007) that prevention-focused individuals are comparatively more attracted to low-power groups than promotion-focused individuals, whereas the reverse is true for high-power groups. Hence, although the high-power group was—in the present research as well as in earlier research using a similar paradigm (Sassenberg et al., 2007, Studies 1–3)—perceived as more attractive than the low-power group, this especially applied to promotion-focused individuals. Importantly, the findings also indicate that prevention-focused individuals do not find low-power groups as unattractive as promotion-focused individuals do, presumably because these groups fit their security-oriented strategies (for evidence, see Sassenberg et al., 2007). Notably, the sample size of the current study is not ideal and, thus, the study is somewhat underpowered. However, this might be considered as a less severe limitation, as the current effect replicates earlier findings (Sassenberg et al., 2007, 2013; Sassenberg and Scholl, 2013).

To extend these findings, Experiment 1B examined how the experience of threat (vs. challenge)—rather than a prevention or promotion focus—predicts individuals’ attraction to group power. Following the design from Experiment 1A, we thus again measured attraction to a high- and low-power group with the identical procedure as before, but this time after inducing a threat vs. challenge state.

## Experiment 1B

### Method

#### Participants and Design

Eighty undergraduates (51 female, 29 male; *M*_age_ = 22.06, SD = 2.00; range 18–27 years) participated in an experiment with a 2 (threat vs. challenge) × 2 (Group power: high vs. low) design. The first factor was manipulated between participants, whereas the second factor constituted a repeated measures factor. Participants were compensated on campus with a candy.

#### Procedure

The procedure of this experiment (from recruiting and location over materials to compensation and debriefing) was exactly the same as in Experiment 1A, except for the fact that the regulatory focus manipulation was replaced by a threat vs. challenge manipulation.

As challenge/threat manipulation, participants completed an open-ended questionnaire on exam preparation. They imagined preparing for an exam in a class that they had already performed once before. They either read that they were dissatisfied with their prior result and were now preparing for a “free-shot” (i.e., the possibility to improve their prior grade; *challenge* condition; *N* = 40) or they read that they had failed the class and were now preparing for their “final chance” (i.e., if they failed again, they would be excluded from the study program; *threat* condition; *N* = 40).^2^

Order of group power was again counter-balanced. There were no interactions with order (i.e., high-power vs. low-power group presented first; all *F*s < 2.17, *p*s > 0.145). The two measures for group attraction showed good internal consistency [2 items each; *r*(80)_high-power-group_ = 0.61, *p* < 0.001; *r*(80)_low-power-group_ = 0.58, *p* < 0.001].

### Results

#### Checks

We first tested if the low-power group was indeed perceived as less powerful than the high-power group. Participants rated the low-power group as having less power (*M* = 3.24, SE = 0.18) than the high-power group (*M* = 7.46, SE = 0.15), *F*(1,77) = 232.23, *p* < 0.001, ${\text{η}}_{\text{p}}^{\text{2}}$ = 0.75. Varying degrees of freedom result from missing data from one participant. There were no interactions of Group power with threat/challenge and/or with Order on perceived power (all *F*s < 1, *p*s > 0.383), indicating that the manipulation of Group power was successful.

#### Attraction to Group Power

We expected that the *low*-power group should be evaluated as even less attractive under threat than challenge, whereas the *high*-power group might be more attractive under threat than challenge.

**FIGURE 2:**
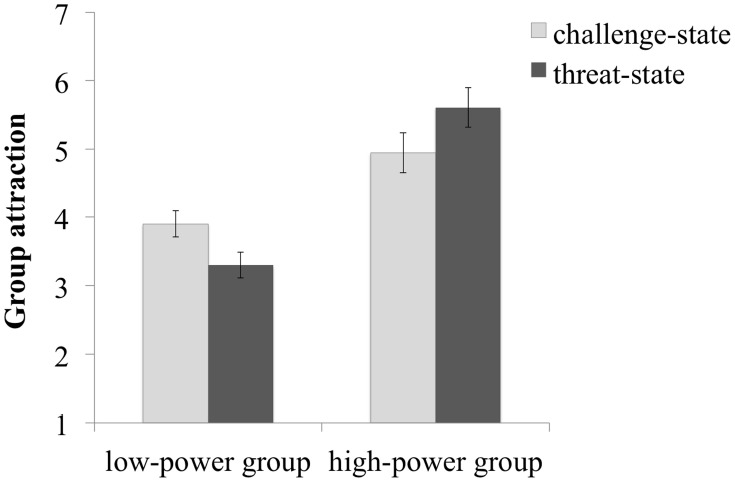
**Group attractiveness depending on group power and threat/challenge state, Experiment 1B (*N* = 80)**.

A 2 (threat vs. challenge) × 2 (Group power: low vs. high) mixed model analysis of variance with repeated measurement on the last factor showed a main effect of Group power, *F*(1,78) = 56.94, *p* < 0.001, ${\text{η}}_{\text{p}}^{\text{2}}$ = 0.42, but not of threat/challenge state (*F* < 1). The high-power group was again perceived as more attractive (*M* = 5.28, SE = 0.20) than the low-power group (*M* = 3.60, SE = 0.14). This effect was qualified by the expected threat/challenge state × Group power interaction, *F*(1,78) = 8.03, *p* = 0.006, ${\text{η}}_{\text{p}}^{\text{2}}$ = 0.09. The high-power group was overall more attractive than the low-power group in the challenge, *F*(1,78) = 11.11, *p* = 0.001, η^2^ = 0.13, and the threat condition, *F*(1,78) = 53.84, *p* < 0.001, η^2^ = 0.41. Supporting our predictions, however, the high-power group tended to be even more attractive under threat (*M* = 5.61, SE = 0.29) than under challenge (*M* = 4.95, SE = 0.29), *F*(1,78) = 2.70, *p* = 0.104, η^2^ = 0.03, MD = 0.66, CI_95%_[–0.14, 1.47], though this effect did not reach conventional levels of statistical significance. In contrast, as predicted, the low-power group was rated as even less attractive under threat (*M* = 3.30, SE = 0.19) than under challenge (*M* = 3.90, SE = 0.19), *F*(1,78) = 4.82, *p* = 0.031, η^2^ = 0.06, MD = 0.60, CI_95%_[0.06, 1.14], see Figure 2.

#### Additional Analyses Combining Data from Experiments 1A and 1B

Taken together, the findings indicated that challenge and prevention focus, as well as threat and promotion focus, yield a similar pattern regarding the attraction to group power. This would result in the prediction of a three-way interaction between the respective Motivational State (regulatory focus vs. challenge/threat), the respective Gain-/Loss-Focus (promotion/challenge: gains; vs. prevention/threat: losses), and Group power (low vs. high). Such an interaction would further support the assumption that in the case of attraction to group power, challenge/promotion focus and threat/prevention focus can have opposite implications, respectively.

To test this, we combined the two data sets from Experiments 1A and 1B, which comprised of the identical dependent measure. A 2 (Motivational State: regulatory focus vs. threat/challenge) × 2 (Gain-/Loss-Focus: gains under promotion/challenge vs. losses under prevention/threat) × 2 (Group power: low vs. high) mixed model analysis of variance with repeated measurement on the last factor was conducted. Please note that in this analysis, the factor Motivational State represents the two separate experiments (Experiment 1A: regulatory focus vs. Experiment 1B: threat/challenge). This analysis showed a main effect of Group power, *F*(1,136) = 72.82, *p* < 0.001, ${\text{η}}_{\text{p}}^{\text{2}}$ = 0.35, which was qualified by the expected three-way interaction, *F*(1,136) = 15.59, *p* < 0.001, ${\text{η}}_{\text{p}}^{\text{2}}$ = 0.10. We explored this 3-way interaction with two separate 2 (Motivational State: regulatory focus vs. threat/challenge) × 2 (Group power: low vs. high) ANOVAs for Gain-/Loss-Focus, respectively.

Indeed, the Motivational State × Group power interaction was significant for gains (promotion vs. challenge), *F*(1,68) = 6.83, *p* = 0.011, ${\text{η}}_{\text{p}}^{\text{2}}$ = 0.09, and also for losses (prevention vs. threat), *F*(1,68) = 9.16, *p* = 0.003, ${\text{η}}_{\text{p}}^{\text{2}}$ = 0.12 (see Table 1, for an overview of all means and SDs).

**TABLE 1:** **Group attractiveness (Means and SDs) depending on group power and motivational state, combined for Experiments 1A and 1B (*N* = 140)**.

		Attraction to low-power group	Attraction to high-power group
Experiment 1A	Promotion	2.95 (1.45)	5.58 (2.01)
	Prevention	3.82 (1.58)	4.58 (1.58)
Experiment 1B	Challenge	3.90 (1.27)	4.95 (2.06)
	Threat	3.30 (1.17)	5.61 (1.50)

There were no interactions between Motivational State and Group power on the attraction to group power or on the power manipulation check, ruling out any differences in the effect of group power between the two experiments. In sum, this indicates that challenge vs. a promotion focus, and threat vs. a prevention focus, differentially predict attraction to a group, depending on this group’s power.

### Discussion

Replicating the previous study, the high-power group was overall more attractive than the low-power group. However, as predicted this pattern depended on individuals’ motivational state: Under challenge, the low-power group was relatively more attractive than under threat. Combined with the results from Experiment 1A, this in sum indicates that for both prevention-focused and challenged individuals, low-power groups are relatively more attractive than for promotion-focused and threatened individuals.

Moreover, the high-power group—on a descriptive level—appeared more attractive under threat than under challenge. Though this pattern has to be treated with caution, it may imply that a high-power group is particularly attractive to promotion-focused (see Experiment 1A) and threatened individuals (more so than prevention-focused and challenged individuals).

## General Discussion

Previous research indicates that threat/challenge and prevention/promotion focus, respectively, are related in several outcome areas, such as avoiding errors/taking advantage of opportunities for gains (Seibt and Förster, 2004; Derks et al., 2006; Oyserman et al., 2007). Based on the example of attraction to group power, the present research indicates that these sets of concepts do, however, differ in the level of evaluating social targets (i.e., evaluating a potential membership in social groups). We predicted this difference between the two sets of states as an exemplary outcome of one fundamental motivational difference between them: the tendency to *engage in* what “feels right” under regulatory focus (Higgins, 2000) and thus sustain the current state, vs. the tendency to overcome a discrepancy by *restoring control* under threat (vs. challenge). On a cautionary note, we investigated these effects in separate studies (rather than one overall study). However, procedures of the studies are highly similar and results from an analysis across both studies confirmed the predicted pattern. Therefore, it is, in our opinion, appropriate to interpret comparisons of results across both studies.

### Implications for Threat/Challenge and Regulatory Focus

Our findings are in line with earlier research on the differential effects of promotion and threat as well as prevention and challenge on attention to negative stimuli (Sassenberg et al., 2015). *Prevention* and *challenge* can lead to similar outcomes (i.e., evaluation of low-power groups and attention to negative stimuli), but other research has also shown that prevention and *threat* are closely related (e.g., Seibt and Förster, 2004; Oyserman et al., 2007). An essential difference between these sets of findings—showing differences or similarities between these motivational states—lies in the *self-relevance* of the assessed outcome.

Studies finding a close relation between prevention and threat (as well as promotion and challenge) mostly test their effect on cognitive styles while processing *neutral* material. Here, threat and a prevention focus result in more local, thorough, and careful information processing. In contrast, challenge and a promotion focus result in more global, flexible, and risky information processing (e.g., Blascovich et al., 1999; Friedman and Förster, 2001). Accordingly, threat/prevention focus and challenge/promotion focus should produce similar responses when *neutral* (i.e., not self-relevant) outcomes are investigated.

In contrast, studies investigating *self-relevant* outcomes—such as perceived valence or the processing of targets otherwise relevant to the self (e.g., group membership, social roles, impressions made on others, performance feedback) appear to produce differential results. Here, promotion and prevention focus direct attention to and foster a positive evaluation of targets that are *congruent* to the judge’s focus: gain-related stimuli in a promotion focus and loss-related stimuli in a prevention focus (see regulatory fit principle; Higgins, 2000). In contrast, threatened individuals direct attention to and foster a positive evaluation of targets that are suitable for (palliative) control restoration: individuals prefer stimuli *opposing* their threat/challenge state (i.e., positive stimuli under threat and negative stimuli when being challenged). Challenged individuals want to master the situation while perceiving sufficient control. Hence, they are ready to “fight barriers” in coping with the demands at hand. Threatened individuals want to restore control and thereby reduce the threat. Thus, resources and opportunities to regain control are particularly attractive. In that sense, the striving to reduce threat is stronger than the search for fitting social context that allow for effortless sustaining of the current strategy. Regulatory focus leads to the striving for fitting behavioral opportunity, whereas threat (and challenge) render the outcomes of choices such as control more relevant.

These explanations on a process level are certainly based on the current data—they are yet speculative and require further research. Nonetheless, the current findings provide evidence for the general idea that promotion/challenge and prevention/threat do not coincide in their effects when it comes to valence outcomes (rather than information processing styles or performance).

### Implications for Threat in Social Interactions

What do the results imply for individual responses to threat in social context? Previous research showed that threat can enhance group cohesiveness (e.g., Sherif and Sherif, 1953; Sherif et al., 1961) and lead to avoidance or rigidity (“freezing”; see Scherer et al., 2004; Mendes et al., 2007), for instance, in terms of adjusting from initial anchors or decisions (Staw et al., 1981; Kassam et al., 2009; Kamphuis et al., 2011; de Wit et al., 2012). Similarly, a related line of research demonstrates that under threat, individuals cling to a high-power *in-group* as a means to restore their control (Fritsche et al., 2008). Extending these findings, our results indicate how individuals under threat (vs. challenge) are attracted to group membership in the first place—that is, to groups they are not yet a member of: threatened individuals strive less for belonging to low-power groups (and potentially more for belonging to high-power groups) than do challenged individuals. They prefer contexts in which they, rather than others, would have control within the social interaction.

Depending on whether the control provided by a high-power group is relevant to the threat at hand or not, striving for membership in such a group could be a comparatively effective strategy to restore control: it could better reduce the stress resulting from the threat than “being frozen” at the status quo. Indeed, the findings contribute to an understanding how the possibility of group membership may serve as a means to regain perceived control under threat in social contexts.

Threat, however, may not only arise on the *individual* level (e.g., in anticipation of an upcoming test), as considered here. It can also result from the *social* context itself—such as when comparing one’s performance with that of superior others, which can threaten one’s self-esteem. This type of threat may likewise determine which group individuals are especially attracted to.

On a more abstract level, the present research indicates that motivational states shape subsequent cognitive evaluations of the social context—here, the valence of group power. As to the impact of threat on social interaction, the current research implies that threatened individuals may seek social roles and social groups that provide them with the means to restore control again. By becoming a member of a high-power group, a threatened individual regains control through being in the dominant social role. Similarly, threat might also lead to a preference for a stable (rather than potentially changing) social role (appointed on a long-term basis rather than elected for a limited time) or for working on an independent (rather than an interdependent) task. These are fruitful approaches for future research which could also be extended beyond the (university) context studied here (e.g., employee samples). In short, threatened individuals (but not those in a prevention focus) seem to prefer social interactions that are predictable (i.e., come with high control).

To conclude, individuals at times face the choice of joining a low-power or a high-power group. While high-power is commonly more attractive than low-power, the respective preference also depends on individuals’ motivation. More specifically, the attraction to a high- and low-power group can depend on whether the group offers the potential to engage in one’s preferred strategies (i.e., fits one’s regulatory focus) or the potential to restore control (i.e., especially under threat) to master the situation at hand and reach a desired end-state. The findings thereby contribute to an understanding why one and the same group may be differentially attractive to be joined, depending on an individual’s current motivational state.

### Conflict of Interest Statement

The authors declare that the research was conducted in the absence of any commercial or financial relationships that could be construed as a potential conflict of interest.

## Footnotes

1 Please note that high-power—as control over own and others’ outcomes—not only implies opportunities, but also a certain responsibility for those others’ situation (see also Chen et al., 2001; Fiske and Berdahl, 2007; Sassenberg et al., 2012). However, at least in Western cultures, individuals spontaneously associate high-power with the opportunities (rather than the responsibilities) it implies (Zhong et al., 2006; Sassenberg et al., 2012). Hence, in the present research, we focus on this predominant understanding of power as providing especially freedom and opportunity (i.e., means to restore control).

2 Notably, the threat manipulation refers to “failure” or “loss,” and the challenge manipulation (indirectly) refers to “gains.” To rule out the possibility that the threat/challenge manipulation had unintentional side effects on participants’ regulatory focus, we pretested the manipulations with a non-overlapping undergraduate sample (*N* = 49; 36 female, 13 male; *M*_age_ = 22.18, SD = 2.94; range 18–31 years). The threat/challenge manipulation neither affected self-reported prevention focus (*M*_challenge_ = 4.58, SD = 1.40; *M*_threat_ = 4.67, SD = 1.23), *t*(47) = 0.24, *p* = 0.405, *d* = 0.07, nor self-reported promotion focus (*M*_challenge_ = 4.99, SD = 0.76; *M*_threat_ = 5.13, SD = 0.52), *t*(47) = 0.79, *p* = 0.216, *d* = 0.22, but it affected self-reported threat (*M*_challenge_ = 4.00, SD = 1.96; *M*_threat_ = 5.00, SD = 1.96; all on 7-point Likert scales), *t*(47) = 1.79, *p* = 0.040, *d* = 0.51 (all one-tailed). Additionally, the threat/challenge manipulation did not affect the number of words participants wrote down in the open-ended questionnaire on exam preparation (*M*_challenge_ = 44.67, SD = 24.63; *M*_threat_ = 42.64, SD = 19.81), *t*(47) = 0.32, *p* = 0.752, *d* = 0.09. This indicates that participants in both conditions exerted a similar level of effort and elaboration while completing the manipulation.
